# Usefulness of Diffusion-Weighted Imaging in Evaluating Acute Cellular Rejection and Monitoring Treatment Response in Liver Transplant Recipients

**DOI:** 10.3390/diagnostics14080807

**Published:** 2024-04-11

**Authors:** Hsien-Jen Chiang, Yi-Hsuan Chuang, Chun-Wei Li, Chih-Che Lin, Hock-Liew Eng, Chao-Long Chen, Yu-Fan Cheng, Ming-Chung Chou

**Affiliations:** 1 Department of Diagnostic Radiology, Kaohsiung Chang Gung Memorial Hospital, Chang Gung University College of Medicine, Kaohsiung 83301, Taiwan; chianghj@adm.cgmh.org.tw (H.-J.C.); jenaqwer@gmail.com (Y.-H.C.); 2 Department of Diagnostic Radiology, Kaohsiung Municipal Feng Shan Hospital—Under the Management of Chang Gung Medical Foundation, Kaohsiung 83062, Taiwan; 3 Graduate Institute of Medicine, College of Medicine, Kaohsiung Medical University, Kaohsiung 80708, Taiwan; 4 Department of Medical Imaging and Radiological Sciences, College of Health Science, Kaohsiung Medical University, Kaohsiung 80708, Taiwan; cwl0331@gmail.com; 5 Liver Transplantation Center, Department of Surgery, Kaohsiung Chang Gung Memorial Hospital, Chang Gung University College of Medicine, Kaohsiung 83301, Taiwan; immunologylin@gmail.com (C.-C.L.); clchen@adm.cgmh.org.tw (C.-L.C.); 6 Department of Surgery, Kaohsiung Municipal Feng Shan Hospital—Under the Management of Chang Gung Medical Foundation, Kaohsiung 83062, Taiwan; 7 Department of Pathology, Kaohsiung Chang Gung Memorial Hospital, Chang Gung University College of Medicine, Kaohsiung 83301, Taiwan; eng6166.hsing@msa.hinet.net; 8 Department of Medical Research, Kaohsiung Medical University Hospital, Kaohsiung 80708, Taiwan; 9 Center for Big Data Research, Kaohsiung Medical University, Kaohsiung 80708, Taiwan

**Keywords:** acute cellular rejection, liver transplantation, diffusion-weighted imaging, apparent diffusion coefficient, immunosuppressant treatment

## Abstract

Acute cellular rejection (ACR) is a significant immune issue among recipients following liver transplantation. Although diffusion-weighted magnetic resonance imaging (DWI) is widely used for diagnosing liver disease, it has not yet been utilized for monitoring ACR in patients after liver transplantation. Therefore, the aim of this study was to evaluate the efficacy of DWI in monitoring treatment response among recipients with ACR. This study enrolled 25 recipients with highly suspected ACR rejection, and all subjects underwent both biochemistry and DWI scans before and after treatment. A pathological biopsy was performed 4 to 24 h after the first MRI examination to confirm ACR and degree of rejection. All patients were followed up and underwent a repeated MRI scan when their liver function returned to the normal range. After data acquisition, the DWI data were post-processed to obtain the apparent diffusion coefficient (ADC) map on a voxel-by-voxel basis. Five regions of interest were identified on the liver parenchyma to measure the mean ADC values from each patient. Finally, the mean ADC values and biochemical markers were statistically compared between ACR and non-ACR groups. A receiver operating characteristic (ROC) curve was constructed to evaluate the performance of the ADC and biochemical data in detecting ACR, and correlation analysis was used to understand the relationship between the ADC values, biochemical markers, and the degree of rejection. The histopathologic results revealed that 20 recipients had ACR, including 10 mild, 9 moderate, and 1 severe rejection. The results demonstrated that the ACR patients had significantly lower hepatic ADC values than those in patients without ACR. After treatment, the hepatic ADC values in ACR patients significantly increased to levels similar to those in non-ACR patients with treatment. The ROC analysis showed that the sensitivity and specificity for detecting ACR were 80% and 95%, respectively. Furthermore, the correlation analysis revealed that the mean ADC value and alanine aminotransferase level had strong and moderate negative correlation with the degree of rejection, respectively (r = −0.72 and −0.47). The ADC values were useful for detecting hepatic ACR and monitoring treatment response after immunosuppressive therapy.

## 1. Introduction

Liver transplantation is a well-established treatment and has become the standard of care for patients with end-stage liver disease [1,2,3,4,5], including living donor liver transplantation (LDLT) and deceased donor liver transplantation (DDLT). In Asia, LDLT is mainly used because this procedure is more acceptable and accounts for 90% of liver transplants; however, in the West, DDLT is the mainstream and accounts for 95% of all liver transplants in the United States [1,2,3]. The one-year patient survival rates for adults and children after primary liver transplantation were 94% and 94.4%, respectively [6], and the short-term patient survival rates were not significantly different between patients treated with LDLT and DDLT [7]. The survival rate following transplantation has significantly improved due to advancements in surgery and the development of immunosuppressive agents [8,9,10]. Acute cellular rejection (ACR) manifests as the sudden decrease of allograft function and inflammation in portal, bile duct, and portal veins, and generally occurs from 5 to 30 days after liver transplantation [11,12,13,14]. ACR is a significant factor that affects the function and survival rate of approximately 15–40% of liver transplantation recipients [15,16]. In clinical, ACR is suspected in patients with liver transplantation when their hepatic enzymes and/or bilirubin levels increase abnormally; however, these biochemical markers are not accurate enough to detect ACR [17]. Liver biopsy is currently the gold standard for diagnosing ACR [18], and the severity of ACR is classified according to the Banff consensus criteria [13]. When patients were diagnosed with ACR, escalation of immunosuppressant dosage or use of steroid bolus was helpful to improve their liver functions without adverse effect on allograft survival rate [19]. However, the invasive biopsy procedure carries risks such as bleeding or infection, so it is not suitable for monitoring treatment response in patients with ACR. Therefore, the development of a non-invasive method for quantifying liver rejection and monitoring treatment response would be highly valuable.

Previous studies have performed non-invasive Doppler ultrasound technique to assess the hemodynamics of allograft for detection of ACR in patients with liver transplantation [20,21,22,23,24,25]. Via Doppler ultrasound, some studies demonstrated that the hemodynamic changes of hepatic artery, portal vein, and splenic artery were helpful for diagnosis of ACR [20,21,22], but others did not find Doppler ultrasound useful for ACR detection [23,24]. The inconsistent results are probably attributable to the fact that Doppler ultrasound assessment is operator-dependent and mainly focused on the hemodynamic changes of liver allograft. In contrast, a previous study further utilized ultrasound transient elastography to assess tissue stiffness in patients with liver transplantation and found that liver stiffness was accurate for diagnosis of ACR when compared with that of healthy subjects [26]. However, the transient elastography was unable to differentiate ACR from hepatitis C, suggesting that transient elastography is not disease-specific and only reflects the stiffness property of tissues.

Contrast-enhanced computed tomography (CT) can provide high-resolution three-dimensional images suitable for morphological assessment in patients with ACR after liver transplantation. One previous study revealed that contrast-enhanced CT was helpful to detect globular swelling, narrowing of hepatic veins, and heterogeneous enhancement of liver parenchyma in ACR patients [27]. However, the results demonstrated that contrast-enhanced CT images were not sensitive to hepatic ACR, probably due to the fact that the CT images provided mainly anatomic information with low soft-tissue contrast. In contrast, nuclear medicine imaging is a functional modality and has previously been performed to assess the graft function and ACR after liver transplantation. One previous study performed ^99m^Tc-labeled diethylenetriaminepentaacetic acid galactosyl human serum albumin (GSA) scintigraphy in patients with liver transplantation, and showed that GSA had the potential for assessing the allograft function non-invasively [28]. In addition, ^99m^Tc hepatobiliary scintigraphy (HBS) can reflect liver uptake and excretion functions and may be useful for diagnosis of ACR after liver transplantation. Because hepatic ACR causes biliary complications along with liver dysfunction, some previous studies performed HBS to assess liver uptake function in patients following liver transplantation. The results demonstrated that the liver uptake function was closely related with histologic findings [29], and that HBS was helpful to distinguish ACR from normal allografts [30,31].

Moreover, magnetic resonance imaging (MRI) is a non-invasive and non-ionizing radiation modality widely used for diagnosis of liver diseases using different pulse sequences [32,33,34,35,36]. Among MRI techniques, diffusion-weighted imaging (DWI) can measure molecular diffusion using apparent diffusion coefficient (ADC) values, whereas a blood oxygenation level-dependent (BOLD) technique can quantify the oxygenation levels using susceptibility-induced relaxation rate (R2*) values. Previous studies have employed DWI and BOLD MRI techniques to detect ACR in liver transplantation patients, and found that the ADC and R2* values were significantly different between patients with ACR and without ACR [37,38,39]. Their findings indicate that ACR patients exhibited hepatic tissue inflammation that caused restriction of diffusion and abnormal increase in hepatic arterial flow due to the increased portal pressure. Thus, both ADC and R2* values serve as reliable biomarkers for ACR detection. In assessment of treatment response, a previous study further demonstrated significantly increased R2* values in ACR patients after immunosuppressant treatment. The results suggested that the portal pressure and hepatic arterial flow were decreased because tissue inflammation was mitigated after the treatment [38].

Although ADC values have been demonstrated useful for detecting ACR, it remains unknown whether ADC values are helpful to monitor treatment response in ACR patients after immunosuppressant treatment. Therefore, we hypothesized that immunosuppressant treatment helps reduce inflammation and increase diffusivity of allograft tissues in ACR patients. The objective of this study was to utilize DWI, biochemistry, and histopathological analysis to evaluate the usefulness of ADC values in monitoring the microstructural alterations in liver tissue of ACR patients after immunosuppressant treatment.

## 2. Materials and Methods

### 2.1. Patients

This prospective study protocol was approved by the Institutional Review Board of Chang Gung Medical Foundation. All participants signed a consent form indicating their agreement to participate in the study, and informed consent was obtained. A total of 25 recipients (22 males and 3 females) with a mean age of 53.6 years and laboratory abnormalities highly suspicious for ACR were enrolled in this study. A pathological biopsy was performed 4 to 24 h after the MRI examination. After confirming the pathology results, ACR patients (*N* = 20) were treated with immunosuppressants, while non-ACR patients (*N* = 5) were treated with routine medication for cholangitis and fatty changes. All patients were followed up regularly with a blood test and Doppler ultrasound examination. The blood test was performed to monitor liver functions, whereas Doppler ultrasound was used to assess the portal and hepatic veins, hepatic arterial flow, and biliary tree. All patients underwent a repeated DWI scan when their liver function returned to the normal range. The mean interval between the two MRI scans was 272.1 days (range: 34–594).

### 2.2. Routine Biomarkers

On the same day before the MRI scans, a 20 mL blood sample was obtained from a peripheral vein to determine liver biochemistry, including aspartate aminotransferase (AST), alanine aminotransferase (ALT), total bilirubin (T-bil), and platelet count (PLT). The blood test was repeated based on clinical status, with intervals adjusted according to clinical treatment needs. In this study, these biomarkers were measured from ACR and non-ACR patients before and after treatment and were used to evaluate their liver functions.

### 2.3. Imaging Data Acquisition

MRI scanning was performed using a 3.0 T MR system (MAGNETOM Skyra, Siemens Medical Systems, Erlangen, Germany) with an 18-channel body matrix coil combined with a spine coil. All patients were required to fast for 4 h before the MR examination. After patients were immobilized in a supine position, they were asked to raise their arms and breathe slowly and smoothly to reduce artifacts during the MRI scans. The liver MRI protocols included tri-planar scans for localization, and axial T1-weighted (in/out phase), T2-weighted (with and without fat saturation), high-resolution three-dimensional T1-weighted images, MR angiography, and cholangiopancreatography scans for diagnosis of tumor recurrence, biliary obstruction, and liver abnormalities. In addition, the DWI pulse sequence was performed with the following imaging parameters: thickness/gap = 5 mm/0 mm; repetition time = 500 ms; echo time = 200 ms; anterior-to-posterior phase encoding direction; acceleration factor = 2; field of view = 500 × 500 mm^2^; number of diffusion direction = 3; number of slices = 32–36; b values = 0 and 800 mm/s^2^; and scan time = 4 min.

### 2.4. Imaging Analysis

**Figure 1:**
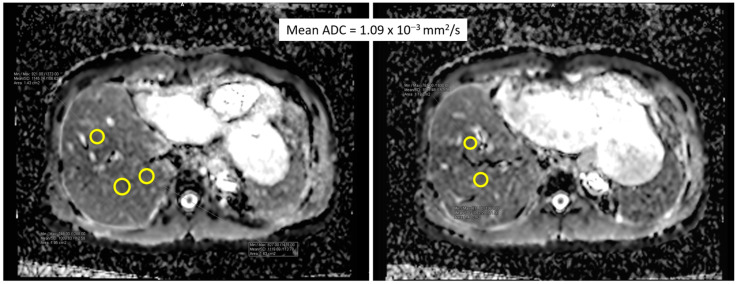
The placement of five regions of interest (yellow circles) on the ADC map for the purpose of measuring the average ADC values of the liver parenchyma.

All DWI data were transferred and post-processed on a dedicated workstation, and ADC maps were calculated based on a mono-exponential model using commercial software (Siemens; Wp workstation package VE11A). Specifically, the ADC value, which reflects motion of water molecules, was derived from the equation $ADC=\ln\left(\frac{S}{S_{0}}\right)/-b$, where S and S_0_ are the signal intensity of DWI and b0 images, respectively, and b is 800 mm/s^2^ in this study. Five regions of interest (ROIs) with an area of 1.5–2 cm^2^ inside the liver graft were placed on ADC maps, focusing on low-signal parenchyma and excluding the uppermost and lowermost parts of the liver, major blood vessels, the hilar area, and the margins of the liver, as shown in Figure 1. Finally, the ADC values of the five ROIs were averaged for statistical analysis. The hepatic ADC values were independently measured by two radiologic technicians (H.J.C. and C.C.L., with 10 and 8 years of experience in MRI examination, respectively) who were blinded to patients’ clinical data.

### 2.5. Histological Diagnosis

After the first MRI examination, all patients underwent core needle biopsy, and the sampling location was determined based on the donated graft. Histological analysis of the specimens was performed by two independent pathologists who evaluated the pathological changes using the Banff criteria rejection activity index (RAI) and diagnosed the severity of acute rejection as mild (3–4), moderate (5–6), or severe (>6). The pathological results were used as a standard to evaluate the performance of ADC values in diagnosing liver ACR and monitoring treatment response.

### 2.6. Statistical Analysis

To assess the inter-observer agreement between two operators and the relationship between liver ADC, biochemistry, and degree of rejection, Pearson’s correlation analysis was performed to calculate correlation coefficients. The correlations were considered weak, moderate, strong, or excellent if the correlation coefficients were 0.3–0.49, 0.5–0.69, 0.7–0.89, or 0.90–1.0, respectively. The Mann Whitney U test was performed to compare ADC values and biochemistry data between ACR and non-ACR patients. Analysis of variance (ANOVA) was used to determine whether hepatic ADC values significantly differed among patients with different pathological grading. Receiver operating characteristic (ROC) curve analysis was performed, and the area under the curve (AUC) was used to evaluate the performance of ADC values in detecting ACR. To understand the treatment response, a Wilcoxon signed-rank test was used to compare the ADC values in ACR and non-ACR patients before and after treatment. All statistical analyses were conducted using IBM SPSS Statistics for Windows (Version 20.0, IBM Corporation, Armonk, NY, USA), and statistical significance was considered if *p* < 0.05.

## 3. Results

Of the 25 patients enrolled, 18 (72%) received LDLT while 7 (28%) received DDLT. Of 18 patients with LDLT, 15 had an allograft of the right lobe while 3 had an allograft of the left lobe. Of 7 patients with DDLT, 6 had an allograft of the whole liver while 1 had an allograft of the right lobe. Based on histopathological results, 20 (80%) recipients had ACR, and 5 (20%) recipients exhibited abnormal liver function due to other liver diseases, such as cholangitis and fatty changes. Among the 20 ACR patients, biopsy results indicated that 10 had mild rejection, 9 had moderate rejection, and 1 had severe rejection according to the Banff rejection index. Table 1 provides the demographic characteristics and histopathological results of the enrolled patients.

In the biochemistry study, there were no significant differences in AST, ALT, T-bil, and PLT levels between the ACR and non-ACR groups before and after treatment. Furthermore, no significant difference was noted between the patients with mild (*N* = 10) and moderate-to-severe (*N* = 10) rejection. In the longitudinal analysis, the Wilcoxon signed rank test showed that immunosuppressive treatment significantly reduced the AST, ALT, and T-bil levels in ACR patients, while the ALT and T-bil levels were significantly decreased in non-ACR patients after treatment, as shown in Table 2. Moreover, the correlation analysis revealed a moderate correlation between ALT levels and degree of rejection (r = −0.47 and *p* = 0.017) in all patients; however, no significant correlation was noted between AST, T-bil, PLT, and degree of rejection.

**Figure 2:**
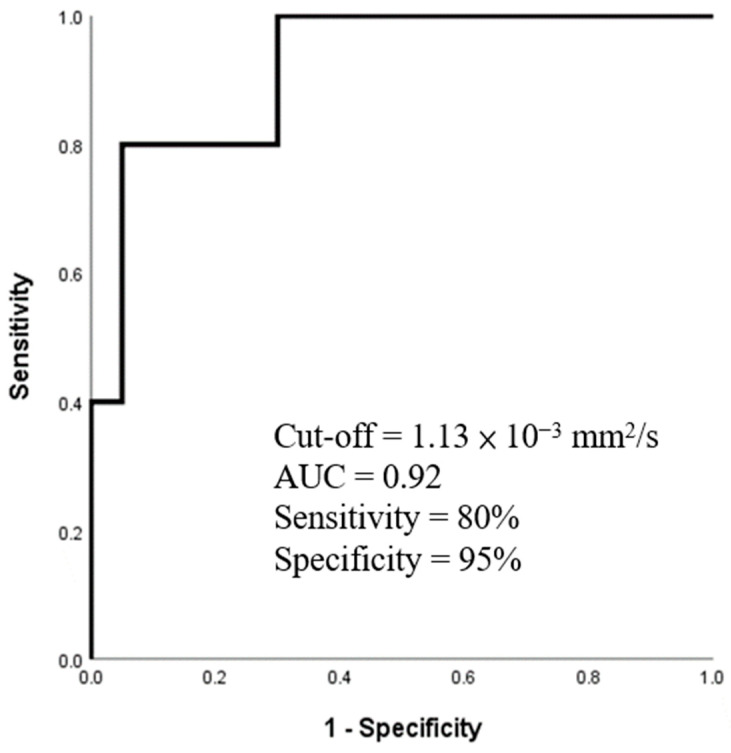
The ROC curve for ADC values in distinguishing ACR and non-ACR patients before treatment.

Regarding the ADC analysis, Pearson’s correlational analysis demonstrated good agreement in ADC measurements between the two operators (r = 0.714). The Mann Whitney U test revealed significantly lower ADC values in ACR patients compared with the non-ACR groups before treatment, as shown in Table 2. The ROC analysis indicated that ADC values had good performance in differentiating between ACR and non-ACR patients (AUC = 0.92 and *p* < 0.002), with a sensitivity of 80% and specificity of 95% (cutoff = 1.133 × 10^−3^ mm^2^/s), as depicted in Figure 2. Moreover, the comparisons further revealed that the ADC values were significantly different between patients with mild (*N* = 10) and moderate-to-severe (*N* = 10) rejection (1.03 ± 0.07 × 10^−3^ mm^2^/s vs. 0.92 ± 0.11 × 10^−3^ mm^2^/s, *p* < 0.05).

**Figure 3:**
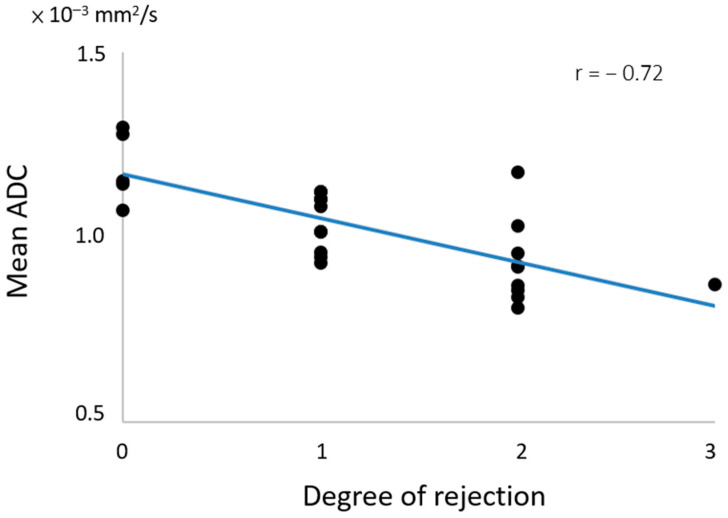
The correlation between mean ADC values and degree of rejection.

In the longitudinal analysis, the Wilcoxon signed-rank test revealed a significant increase in ADC values in the two groups after treatment; however, no significant difference in ADC values was observed between the two groups after treatment. Furthermore, ANOVA indicated a significant change in ADC values with different degrees of rejection (*p* < 0.05), and correlation analysis demonstrated a significantly negative strong correlation between mean ADC value and degree of rejection (r = −0.72 and *p* = 0.000), as shown in Figure 3. In biochemistry, there was a significantly negative moderate correlation between ALT and degree of rejection (r = −0.47 and *p* = 0.017). However, no significant correlation was noted between ADC values and liver function (i.e., ALT, AST, T-bil, and PLT).

## 4. Discussion

Liver transplantation is an effective way to prolong the life of patients with end-stage liver disease, with 5-year survival for over 70% of recipients [40,41,42]. While appropriate immunosuppression has reduced the risk of ACR, it remains the leading cause of graft dysfunction, occurring in about 20–70% of all transplant patients [43,44,45,46,47]. Therefore, monitoring the treatment response is crucial in managing ACR patients to reduce their morbidity and mortality. One previous study performed BOLD MRI to monitor treatment response in patients with ACR, and demonstrated that the hepatic R2* values were significantly increased after immunosuppressant treatment. The findings indicated that the treatment reduced the inflammation and decreased the portal pressure, decreasing the hepatic arterial flow into the liver [38]. However, no previous study has performed DWI to directly measure the microstructural diffusion and monitor the treatment response in patients with ACR.

The present study performed repeated DWI scans to evaluate the microstructural alterations of liver tissue in ACR and non-ACR patients before and after immunosuppressant treatment. Our results demonstrated that the ADC values and liver function were significantly lower in ACR patients than those of non-ACR patients and that the ADC values had high performance in the detection of ACR with AUC = 0.92. After treatment, the ADC values and liver function significantly increased in the ACR patients to values close to those of non-ACR patients with treatment. These findings suggest that DWI is an accurate technique for detecting ACR and suitable for monitoring the treatment response in patients with ACR.

Histopathological evaluation of liver biopsies is currently considered the “gold standard” for diagnosing graft dysfunction following liver transplantation [11,12,48]. Hepatic ACR occurs when recipient T cells recognize the donor allograft antigen, leading to a cytopathic immune response against the donor tissues [16,49]. The severity of rejection is classified according to the Banff consensus criteria [13], and the RAI is based on scoring features in three categories: portal inflammation, bile duct inflammation damage, and venous endothelial inflammation. These features include lymphocyte infiltration, fibrous proliferation, bile duct hyperplasia, or tissue edema [13,48,50]. These pathological changes are closely associated with tissue inflammation, leading to a decrease in water molecule diffusion within the allograft tissues due to reduced extracellular space. Over time, this process may eventually contribute to the development of liver cirrhosis [51,52,53].

DWI is a suitable method for quantitatively measuring molecular diffusion and has been widely used to investigate micro-architectural changes in biological tissues. In line with a previous study [37], our present study demonstrated that the ADC values in the ACR group were significantly lower compared with the non-ACR group. These findings indicate that ACR leads to portal inflammation with increased infiltration of lymphocytes, eosinophils, neutrophils, and macrophages, directly impeding the mobility of water molecules. However, no significant difference was noted in liver functions between the two groups before and after treatment, suggesting that the conventional biochemistry was not sensitive enough to detect hepatic ACR. Furthermore, we found a negative moderate correlation between the ALT and the degree of rejection (r = −0.47), and a negative strong correlation between the ADC and the degree of rejection (r = −0.72). The results indicate that the ADC value is a more suitable biomarker than biochemistry for reflecting the severity of rejection in ACR patients.

Following immunosuppressant treatment, one previous study using BOLD MRI revealed a significant increase in R2* values in ACR patients, indicating a reduction in portal pressure and an increase in hepatic arterial flow to the liver [38]. Additionally, our present study demonstrated a significant elevation in ADC values in the allograft tissues of ACR patients after treatment. Collectively, these findings suggest that increased ADC values are likely to be attributable to reduced lymphocyte infiltration, fibrous proliferation, bile duct hyperplasia, and tissue edema, as well as increased perfusion from the hepatic artery following immunosuppressant therapy. However, further investigation will be needed to demonstrate the histopathological changes in ACR patients before and after immunosuppressant treatment.

Many studies have shown that ADC values were decreased in liver parenchyma of patients with non-alcoholic fatty liver disease, steatosis, and liver cirrhosis [54,55,56]. Regarding liver transplantation, Sandrasegaran et al. performed DWI to evaluate parenchymal disorders in patients after orthotopic liver transplantation (OLT) and found that differences in ADC values were associated with a histological abnormality [39]. In addition, they suggested that an ADC value of <0.99 × 10^−3^ mm^2^/s was suitable for predicting a parenchymal histological abnormality in patients after OLT (AUC = 0.84, sensitivity = 0.85, and specificity = 0.72). Lin et al. performed DWI with multiple b-value acquisition to assess graft rejection in patients with liver transplantation, and found that DWI with a high b-value of 600 or 800 s/mm^2^ was suitable for diagnosis of ACR in patients after liver transplantation (AUC = 0.784 and 0.861, respectively) [37]. The present study performed DWI with a high b-value of 800 s/mm^2^ for detecting ACR and further monitoring treatment response in patients with and without ACR. Our results demonstrated that the ADC values were accurate in detection of ACR in recipients, and an ADC value of <1.133 × 10^−3^ mm^2^/s was suitable for detecting ACR (AUC = 0.92, sensitivity = 80%, and specificity = 95%). However, the discrepancies in the cutoff ADC values between previous studies and ours might be attributable to graft type, whole liver, and partial lobe liver transplantation.

There are several limitations of this study that warrant discussion. First, the sample size was small because the patients needed to undergo repeated MRI scans and biochemistry studies before and after immunosuppressant treatment. Second, this study did not include a repeated biopsy for the patients after the treatment because the biopsy is an invasive procedure with risk of bleeding and infection. Third, this study enrolled only one patient with severe ACR. A study with more patients with severe ACR will be needed to further understand the relationship between rejection severity and ADC values. Finally, the cutoff ADC value determined in the present study was based on patients with highly suspected ACR, so it may not be sensitive for detecting subtle ACR in patients with liver transplantation.

## 5. Conclusions

In conclusion, our study utilized liver DWI, biochemistry, and histological analysis to investigate the differences in ADC values and biochemical data between ACR and non-ACR groups before and after treatment. The findings revealed that the ADC value served as a reliable imaging biomarker for distinguishing between ACR and non-ACR patients prior to treatment. Furthermore, the immunosuppressive treatment significantly elevated the ADC values and improved liver functions in ACR patients, bringing them to levels comparable to those of non-ACR patients. Based on these results, we conclude that DWI-derived ADC values hold promise for assessing hepatic ACR and monitoring treatment response following immunosuppressive therapy.

## Figures and Tables

**Table 1:** The demographic characteristics and histopathological results in the enrolled patients with liver transplantation.

Total *n* = 25	
Male = 22	
Female = 3	
Mean age (range) = 53.56 (20–68 years)	
Graft type	
LDLT *n* = 18	
	Right *n* = 15
	Left *n* = 3
DDLT *n* = 7	
	Whole liver *n* = 6
	Right *n* = 1
Histopathology *n* = 25	
Rejection *n* = 20	Non-rejection *n* = 5
Mild *n* = 10	Cholangitis *n* = 3
Moderate *n* = 9	Cholangitis + mild (15%) fatty change *n* = 1
Severe *n* = 1	Minimal (<5%) fatty change *n* = 1
Repeated MRI *n* = 25 Mean (range) interval between two MRI scans = 128 days (33–247 days)	

LDLT: living donor liver transplantation; DDLT: deceased donor liver transplantation.

**Table 2:** Comparison of liver ADC value and biochemical markers in patients with and without rejection before and after treatment.

Rejection (*n* = 20)	Pre-Treatment	Post-Treatment	* p * Value
ADC (10^−3^ mm^2^/s)	0.981 ± 0.109 *	1.300 ± 0.199	0.001
AST (U/L)	544.8 ± 693.5	48.25 ± 38.9	0.000
ALT (U/L)	604.8 ± 393.8	52.6 ± 37.3	0.000
T-bil (mg/dL)	2.0 ± 2.0	1.2 ± 0.9	0.006
PLT (1000/ μ L)	149.8± 73.5	139.8 ± 46.5	0.267
** Non-rejection (*n* = 5) **			
ADC (10^−3^ mm^2^/s)	1.182 ± 0.105 *	1.346 ± 0.169	0.043
AST (U/L)	203.60 ± 121.80	42.2 ± 20.9	0.080
ALT (U/L)	416.20 ± 307.33	46.6 ± 24.7	0.043
T-bil (mg/dL)	3.50 ± 3.46	1.7 ± 1.6	0.006
PLT (1000/ μ L)	119.6 ± 50.6	142.4 ± 51.9	0.138

ADC: apparent diffusion coefficient; AST: aspartate aminotransferase; ALT: alanine aminotransferase; T-bil: total bilirubin; PLT: platelet count. Asterisks (*) indicate significant difference between the rejection and non-rejection groups.

## Data Availability

Data available on request due to ethical and privacy issues.
